# Moonlighting Proteins Are Important Players in Cancer Immunology

**DOI:** 10.3389/fimmu.2020.613069

**Published:** 2021-01-18

**Authors:** Annalisa Adamo, Cristina Frusteri, Maria Teresa Pallotta, Tracey Pirali, Silvia Sartoris, Stefano Ugel

**Affiliations:** ^1^ Section of Immunology, Department of Medicine, University of Verona, Verona, Italy; ^2^ Department of Experimental Medicine, University of Perugia, Perugia, Italy; ^3^ Department of Pharmaceutical Sciences, University of Piemonte Orientale, Novara, Italy

**Keywords:** moonlighting proteins, tumor, immune system, cancer immunomodulation, cancer immunology

## Abstract

Plasticity and adaptation to environmental stress are the main features that tumor and immune system share. Except for intrinsic and high-defined properties, cancer and immune cells need to overcome the opponent’s defenses by activating more effective signaling networks, based on common elements such as transcriptional factors, protein-based complexes and receptors. Interestingly, growing evidence point to an increasing number of proteins capable of performing diverse and unpredictable functions. These multifunctional proteins are defined as moonlighting proteins. During cancer progression, several moonlighting proteins are involved in promoting an immunosuppressive microenvironment by reprogramming immune cells to support tumor growth and metastatic spread. Conversely, other moonlighting proteins support tumor antigen presentation and lymphocytes activation, leading to several anti-cancer immunological responses. In this light, moonlighting proteins could be used as promising new potential targets for improving current cancer therapies. In this review, we describe in details 12 unprecedented moonlighting proteins that during cancer progression play a decisive role in guiding cancer-associated immunomodulation by shaping innate or adaptive immune response.

## Introduction

The ability to induce a host immune response to defeat cancer cells and achieve a durable response has completely changed the paradigm of clinical practices in oncology (1). For instance, the use of immune-checkpoint inhibitors in melanoma or the adoptive therapy based on engineered T-cells in patients with B-cell acute lymphoblastic leukemia has strongly decreased the mortality rate of such incurable pathologies (2). Nevertheless, the existence of conserved mechanisms of immune escape, which are exploited by cancer cells, is now the major limit to successfully build up immunotherapeutic approaches. Indeed, today a substantial proportion of cancer patients do not have benefit from these approaches (2). Nowadays, it is therefore mandatory to define some key features of the interplay between cancer and immune system, unveiling (i) the molecular mechanisms exploited by cancer cells to evade anti-cancer immune responses, (ii) the key players during the events of immunomodulation, and (iii) specific prognostic factors to stratify the patients as responders or non-responders, in order to develop an effective personalized cancer immunotherapy.

**Figure 1:**
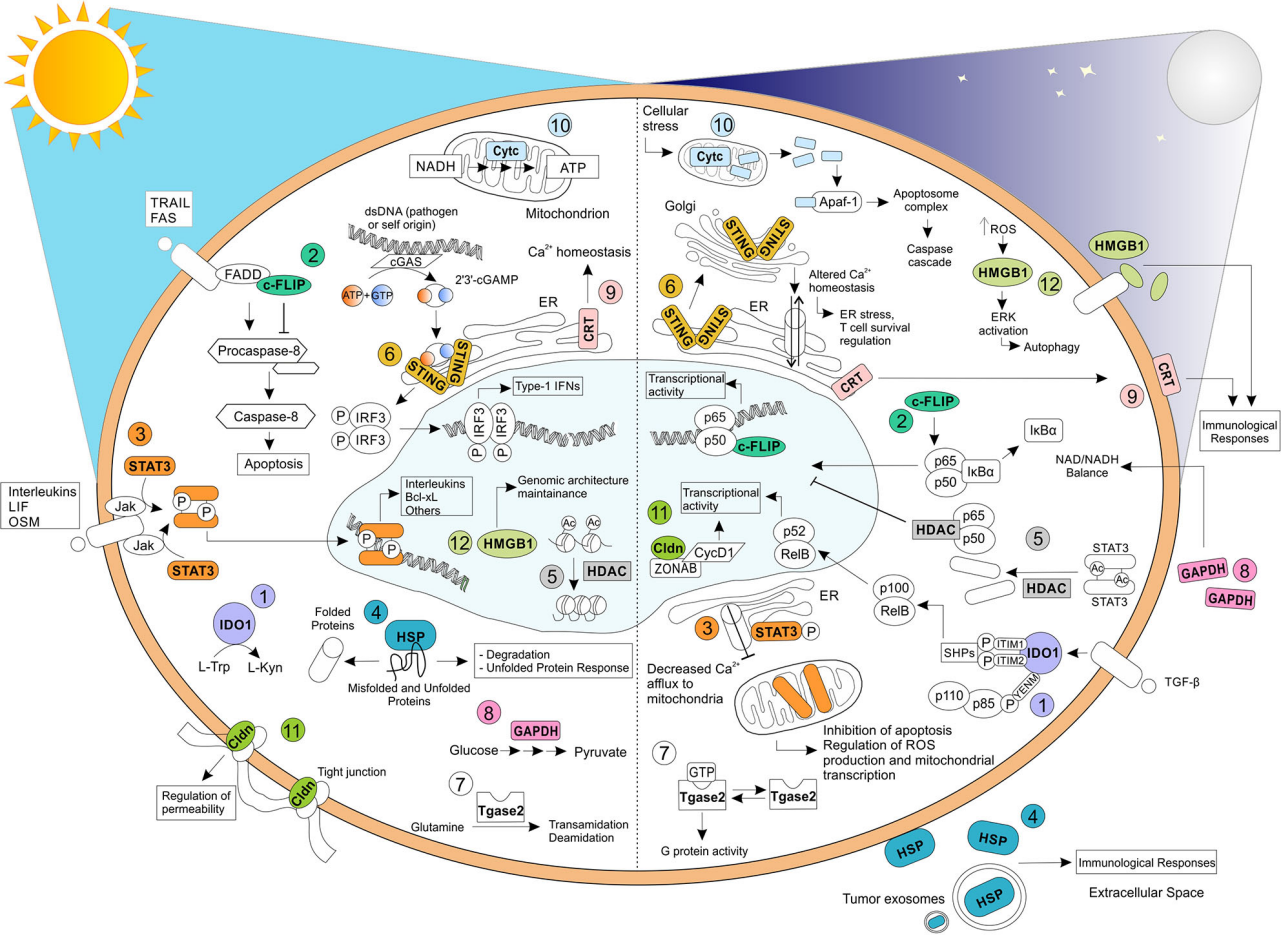
Relevant biochemical and biological functions exhibited by moonlighting proteins. Moonlighting proteins have been reported to exhibit multiple functions, profoundly affected by their cellular localization and environmental stimuli. Here, we have reported, on the left, the well-known and original functions carried oud by (1) IDO1, (2) c-FLIP, (3) STAT3, (4) HSP, (5) HDAC, (6) STING, (7) Tgase2, (8) GAPDH, (9) CRT, (10) Cytc, (11) Cldn, and (12) HMGB1. On the right, some of the apparently unrelated functions mediated by the same proteins have shown. TRAIL, TNF-related apoptosis-inducing ligand; FAS, Fas Cell Surface Death Receptor; LIF, Leukemia inhibitory factor; OSM, oncostatin-M; FADD, Fas-associated protein with death domain; IRF3, Interferon regulatory factor 3; Apaf-1, apoptotic protease activating factor-1; ZONAB, ZO-1–associated nucleic acid binding protein; CycD1, Cyclin D1.

Recently, well-known proteins have been reported to exploit novel activities, apparently unrelated to the original functions the expression “moonlighting proteins” has been coined (3, 4). These findings have partially changed our understanding of cell biology. Indeed, the localization of a protein in a new cellular environment could promote unpredicted molecular interactions by many potential new binding partners and contribute to generate new functions. Moreover, multifunction proteins might represent an original mechanism exploited by cells to overcome their limited amount of genomic information (5). Many reports have demonstrated that moonlighting proteins use their canonical biology and binding sites for carrying out their unexpected activities (5–7). However, emerging studies have unveiled also the presence of different active structure domains responsible for the induction of non-canonical activities (8) especially during pathological condition such as in cancer setting (9). This review aims at a focus analysis of several moonlighting proteins providing the molecular mechanisms they use to modulate the immune response in cancer. In particular, we outline 12 conventional proteins mainly expressed by immune cells that operate as metabolic enzymes (e.g. IDO1, GAPDH), apoptosis-related proteins (e.g. c-FLIP, Cytochrome c), protein sensors (e.g. STING), transcriptional factors (e.g. STAT3), mediators of cell-cell adhesion (e.g. Claudins) as well as structural complex components that maintain genomic stability (e.g. HMGB1) or regulate protein-folding and cell-signaling pathways (e.g. Hsps, HDAC, Calreticulin, Tgsa2) at the steady state but, during cancer progression, they acquire unpredictable functions in controlling the crosstalk between cancer and immune system (**Figure 1**). Since these putative moonlighting proteins manipulate the anti-tumor immune response, they can be enlisted as potential targets for cancer immunotherapy.

## Indoleamine 2,3-Dioxygenase

Indoleamine 2,3-Dioxygenase 1 (IDO1) represents one of the most interesting moonlighting proteins linking an ancient metabolic pathway with immune regulation. IDO1 is a heme-containing enzyme that catalyzes the oxidative cleavage of the indole ring in L-tryptophan, regulating the catabolism of this essential amino acid at the initial, rate-limiting level in a specific pathway. This activity causes the production of kynurenine, which represents the upstream metabolite along the so-called kynurenine pathway (10). The definition of the crystal structure of human IDO1 has revealed a folding into a catalytic large C-terminal domain containing the heme, a non-catalytic small N-terminal domain, and a long loop connecting the two domains (11). IDO1’s metabolic function was initially thought to mediate a survival strategy to deprive bacteria and cancer cells of tryptophan (12). The discovery that the catabolism of this essential amino acid was crucially involved in maintaining maternal T cell tolerance at the maternal-fetal interface greatly raised the interest in IDO1 and a very fruitful area of study was opened (13). Now IDO1 is considered as an authentic immune regulator capable of fine-tuning both innate and adaptive immune responses under a variety of conditions, ranging from pregnancy (13) and transplantation (14) to infection (15), chronic inflammation (16), autoimmunity (17), and neoplasia (18). IDO1 immunoregulatory effects are mainly mediated by dendritic cells (DCs), the most potent antigen presenting cells that, upon IDO1 upregulation, acquire tolerogenic functions. Its enzymatic activity causes tryptophan depletion in local tissue microenvironments and formation of bioactive metabolites collectively called kynurenines. As a result, IDO1-expressing DCs mediate multiple effects on T lymphocytes, including inhibition of proliferation, apoptosis, and differentiation towards a regulatory phenotype (19, 20). These effects are induced by interferon (IFN)-γ, which activates intense but short-term IDO1-mediated immunosuppressive activity related to the enzymatic function of IDO1. In fact, tryptophan starvation in DCs expressing functional IDO1 causes accumulation of uncharged transfer RNA (tRNA) in neighboring T cells, that represent the signal for activation of the amino acid-sensitive general control non-derepressible 2 (GCN2) stress-kinase pathway (21, 22). GCN2 initiates a stress-response program that triggers cell-cycle arrest and anergy induction in responding T cells, but also causes the blocking of the conversion of regulatory T cells (Tregs) into Th17-like cells and the downregulation of T-cell receptor ζ-chain (TCRζ) in CD8^+^ T cells (23, 24). In macrophages the IDO1-GCN2 pathway is responsible of an immunosuppressive phenotype, with increased production of interleukin (IL)-10 and transforming-growth factor (TGF)-β (25). Kynurenine regulates immune homeostasis by acting as a ligand of Aryl hydrocarbon Receptor (AhR) in both T cells and DCs. In particular, AhR activation promotes conversion of effector T lymphocytes into Tregs and upregulates IDO1 expression in DCs, further amplifying immunoregulatory effects and blocking anti-tumor immunity (26–28). These DCs increase the production of the anti-inflammatory cytokines TGF-β1 and IL-10, and lower the production of pro-inflammatory cytokines, such as IL-1β (29). Kyn-mediated activation of AhR induces a tolerogenic phenotype also in macrophages, by regulating the expression of immunosuppressive molecules such as programmed death-ligand 1 (PD-L1) (30). Such mechanisms are exploited by tumors to escape immunosurveillance. In fact, IDO1 is expressed in immune cells and in tumor cells themselves (31). In 2011, it was discovered that IDO1 does not merely degrade tryptophan and produce kynurenine, but it also acts as a signal-transducing molecule (32, 33) (**Table 1**). IDO1’s non-enzymic function, namely, intracellular signaling events, relies on the presence of two immunoreceptor tyrosine-based inhibitory motifs (ITIMs) in the non-catalytic, small domain of IDO1. Interestingly, the paralogue of IDO1, IDO2, which is considered to be the ancestral form of IDO1 (also known as proto-IDO, being expressed also in prokaryotes and lower vertebrates) (35), contains only one functional ITIM and does not transduce signals (32). IDO1 signaling activity is triggered in DCs by TGF-β (36), which promotes IDO1’s ITIMs phosphorylation by tyrosine kinase of the Src family, specifically, Fyn in plasmacytoid DCs (pDCs) (32) and Src in conventional DCs (cDCs) (27, 34). As a consequence, these domains act as docking sites for the tyrosine phosphatases SHP-1 and SHP-2, which directly interact with IDO1 and activate signaling events leading to activation of the non-canonical NF-κB pathway and upregulation of both IDO1- and TGF-β–encoding genes (32, 34). The gene expression reprogramming promotes the induction of a stable regulatory phenotype in both pDCs and cDCs. In contrast, in a microenvironment dominated by the presence of the pro-inflammatory cytokine IL-6, IDO1’s half-life is shorted. In fact, suppressor of cytokine signaling 3 (SOCS3) can bind phosphorylated ITIMs and leads to ubiquitination and subsequent proteasomal degradation of IDO1 by recruiting members of the E3 ubiquitin ligase complex (37). In particular, ITIM1 is mostly involved in the partnership with SHPs and ITIM2 is more effective at binding SOCS3 (38). Very recently, it was demonstrated that in addition to ITIMs, IDO1 contains a YxxM (where “x” indicates any amino acid) motif, namely YENM. When phosphorylated, this domain becomes responsible for the direct binding of the class IA PI3K regulatory subunit, p85, and for the consequent activation of p110 subunits. These events lead to IDO1 anchoring to early endosomes (EE) and to the activation of the ITIMs-mediated immunoregulatory IDO1 pathway in pDCs (39). Based on available information, IDO1 may thus represent one of the most important moonlighting proteins in immune regulation. In fact, IDO1 (i) is a heme-containing enzyme, its catalytic activity is more intense but transient (32) and resides in the cytosol of cells, including DCs and tumor cells; (ii) is a signal transducing molecule that act through phosphorylated ITIMs when anchored to EE, a localization reached from the partnership with, and activation of, class IA PI3K subunits. The design and development of potent and selective inhibitors of the catalytic activity of IDO1 have so far represented the major goal of medicinal chemists in order to enhance anti-tumor immune responses. However, one of these drugs (epacadostat) recently showed poor efficacy in clinical trials on cancer patients (40) and, as a consequence of this failure, other clinical trials with IDO1 inhibitors have been suspended, canceled, or downsized. It is likely that considering IDO1 as a moonlighting protein and targeting its signaling functions may provide much more benefit for successful immunotherapeutic maneuvers in neoplasia.

**Table 1:** IDO1-mediated immunoregulatory functions.

Indoleamine 2,3-Dioxygenase 1 (IDO1)			
IECs	Biological functions	Hypothesized mechanism	Reference
DCs	↑ Tolerogenic functions ↑ Amplification of immunosuppressive effects ↑ Amplification of immunosuppressive effects	Enzymatic activity leading to tryptophan depletion and accumulation of bioactive metabolites in local tissue microenvironment IDO1 upregulation induced by AhR and accumulation of bioactive metabolites TGF-β-mediated IDO1 signaling activity due to ITIMs phosphorylation and non-canonical NF-kB pathway activation	(19) (26–28) (27, 34)
pDCs	↑ Stable immunoregulatory phenotype	TGF-β-mediated IDO1 signaling activity due to YENM activation of class IA PI3K p110 subunits	(27, 32)
T cells	↓ Proliferation ↓ Apoptosis ↓ Differentiation ↑ Anergy ↓ Adaptation ↓ Conversion of Treg into Th17-like cells ↑ Tregs induction	Mechanisms mediated by IDO1-expressing DCs IFN-γ-mediated immunosuppressive activity leading to tRNA uncharged accumulation and GCN2 stress-kinase pathway activation tRNA uncharged accumulation and GCN2 stress-kinase pathway activation tRNA uncharged accumulation and GCN2 stress-kinase pathway activation AhR activation induced by kynurenine	(20–22) (23) (26, 28) (28)

## Cellular Flice (FADD-Like IL-1β-Converting Enzyme)-Inhibitory Protein

Cellular FLICE (FADD-like IL-1β-converting enzyme)-inhibitory protein (c-FLIP) is a crucial anti-apoptotic protein. c-FLIP has 13 distinct spliced variants, three of which are expressed as proteins: the 55 kDa long form (c-FLIP_L_), the 26 kDa short form (c-FLIP_S_), and the 24 kDa form of c-FLIP (c-FLIP_R_) (41). In the tumor context, c-FLIP acts as a drug resistance factor able to suppress cytokine- and chemotherapy-induced apoptosis by interacting with the death signaling complex downstream of tumor necrosis factor (TNF)-α receptors, Fas (CD95), and TNF-related apoptosis inducing ligand (TRAIL) receptors 1 (DR4) and 2 (DR5) (42). In addition to its anti-apoptotic role, c-FLIP plays other key process governing cell survival and death, such as programmed necroptosis and autophagy (41). Indeed, necroptosis is led on the building of a protein complex defined as ripoptosome, which is a signaling complex containing receptor-interacting protein 1 (RIP1), Fas-associated death domain (FADD), caspase-8, caspase-10, and both c-FLIP_L_ and c-FLIP_S_ isoforms. In this context, c-FLIP_L_ has been reported to prevent the ripoptosome formation, whereas c-FLIP_S_ promotes ripoptosome assembly. Therefore, c-FLIP isoforms are involved in switching apoptotic and necroptotic cell death (43). Moreover, c-FLIP_L_ also reduces the autophagy by preventing Atg3 E2 enzyme binding to the microtubule-associated protein 1 Light Chain 3 (LC3) ubiquitin-like protein, a key process upstream of autophagic vesicle expansion (44, 45). In addition to support resistance of cell death, c-FLIP triggers both epithelial-mesenchymal transition (EMT) and motility of cancer cells, thus promoting tumor invasive potential. Therefore, it is unsurprising that high levels of c-FLIP has been reported in several cancer settings such as colorectal cancer (46), cervical cancer (47), pancreatic cancer (48), lung cancer (49), breast cancer (50), Burkitt’s lymphoma (51), and non-Hodgkin’s Lymphoma (52) as well as that patients with tumors expressing high levels of FLIP tend to have a poorer prognosis (53, 54). However, c-FLIP performs also unexpected functions during cancer progression. Indeed, the expression of c-FLIP is constitutively required for the development and survival of immunoregulative cell populations, such as regulatory T cells (Treg) and monocytic myeloid-derived suppressor cells (MDSCs), thus leading to the suppression of the anti-tumor immune response (53, 55, 56) (**Table 2**). Plaza-Sirvent et al. demonstrated that Treg-specific deletion of c-FLIP in mice resulted in a fatal autoimmune disease characterized by the loss of peripheral Tregs and a general hyperactivation of the immune responses (56). Surprisingly, blocking CD95L did not rescue Treg survival *in vivo*, suggesting further c-FLIP functions in Treg cells beyond blocking CD95-mediated apoptosis (56). Furthermore, enforced c-FLIP expression has shown to upregulate several molecules related to the immunomodulation in human monocytes, including IDO1, prostaglandin-endoperoxide synthase 2 (PTGS2), PD-L1, PD-L2, and IL-10, suggesting a MDSC-like profile (53). Fiore et al. also showed that the transcriptional and signaling activity mediated by c-FLIP in myeloid cells was strictly associated to the activation of NF-κB pathway (53). More interestingly, c-FLIP not only promoted the nuclear translocation of the NF-κB subunits p65 and p50, but it directly translocated to nucleus where co-localized with p50 (53). According to these findings, other studies demonstrated the ability of c-FLIP to bind and activate NF-κB in specific immune cell subsets (60). NF-κB activation depends on the formation of a multiprotein complex comprising TNF-receptor-associated factors (TRAFs), NF-kappa-B-inducing kinase (NIK), IκB Kinase -α and -β (IKKα, IKKβ), NF-kappa-B essential modulator (NEMO), inhibitor of NF-κB -α and -β (IκB-α, IκB-β), and IKK complex-associated protein (IKAP), resulting in the phosphorylation and degradation of IκB-α and the release of NF-κB for its nuclear translocation (62). Kataoka and colleagues demonstrated also the ability of c-FLIP_L_ to activate directly both NF-κB and ERK (extracellular-signal-regulated kinase) signaling pathway in lymphocytes (57). Indeed, FasL-treated resting T cells were not only resistant to apoptosis, but they also showed an increased NF-κB- and activator protein 1 (AP-1) -mediated proliferation following CD3 co-stimulation, thus suggesting an additional active role of c-FLIP in T cell signaling beyond the one as an inhibitor of apoptosis. Hence, the authors suggested a model whereby c-FLIP activity mediated by Fas and FADD leads to the recruitment of proteins involved in different signaling pathways, including TRAF1, TRAF2, RIP, and proto-oncogene serine/threonine-protein kinase (Raf-1) to the Fas signaling complex (57). Moreover, the constitutive expression of the c-FLIP_L_ in primary mouse T cells not only conferred protection from FasL-induced cell death, but also decreased the threshold for TCR-triggered proliferation and IL-2 production following ERK and NF-κB activation (58). Therefore, transgenic c-FLIP expression in lymphocytes resulted in increased T cell proliferation and IL-2 production after *in vitro* stimulation with low concentrations of antigens or anti-CD3 (58). In addition, the priming of T cells with PI3K (Phosphatidylinositol 3-kinase) has been reported to facilitate the c-FLIP-dependent ERK activation and IL-2 production, suggesting that the presence of PI3K signaling may convert c-FLIP from an inhibitory to a stimulatory molecule (63). However, the role of c-FLIP on T cell activation is not completely clarified yet. More recently, Koenig and co-authors reported that in T cells c-FLIP_L_ can also heterodimerize with caspase-8 with a death receptor ligation-independent mechanism leading to the activation of caspase-8 mediated by the C-terminus portions of c-FLIP_L_. This interaction induces the cleavage of c-FLIP_L_ at Asp376 by caspase-8 to produce p43FLIP, resulting as stabilizer of caspase-8 activity and promoting the activation of pathways involved in T cell growth (59). Indeed, the acute loss of c-FLIP in effector T cells leads to reduced caspase-8 activity and impairment of cell growth, whereas p43FLIP can rescue T cell survival and growth from the loss of c-FLIP by maintaining caspase-8 in an active form (59). In addition to its first described function as an inhibitor of caspase-8 activation by competitive binding to FADD following death receptor ligation, c-FLIP_L_ is now emerging as a potential activator of caspase-8 and, potentially, its initial substrate. Furthermore, p43FLIP has been already described to associate with Raf1, TRAF2, and Receptor-interacting serine/threonine-protein kinase 1 (RIPK1), thus inducing T cell proliferation (59). Recent studies demonstrated that the cytoplasmic NH2-terminal procaspase-8 cleavage product of c-FLIP (p22-FLIP) expressed in malignant cells, primary T and B cells, and mature DCs strongly induces NF-κB activity by interacting with the IKK complex *via* the IKKγ subunit (60, 64). Baratchian et al. proposed a model of how c-FLIP activate NF-κB showing that cFLIP_L_, cFLIP_S_, and their proteolytic product p22-FLIP require the C-terminal region of NEMO/IKKγ (amino acids 272–419) and its ubiquitin binding function for activating the IKK kinase. Indeed, none of the c-FLIP isoforms created a stable complex with IKKγ, but, on the contrary, all c-FLIP isoforms required the kinase TAK1 (Mitogen-activated protein kinase kinase kinase 7, MAP3K7) to induce IKK kinase activation (61). Notably, c-FLIP_S_ and p22-FLIP have been reported as components of the complex that incorporate FADD and RIP1. Ubiquitinated RIP1 may be generated and then released from such protein complex. RIP1 could directly bind the ubiquitin binding domain of IKKγ, recruiting TAK1, which in turn activates IKK (61). c-FLIP_L_ seems to require the linear ubiquitination complex (LUBAC) to generate an unidentified different ubiquitinated substrate, responsible for IKK activation (61). Therefore, even if the molecular mechanisms remain partially unknown, c-FLIP clearly exhibits moonlighting functions on modulating activities and functions of immune cells during cancer progression, thus representing a potential target for developing more effective cancer immunotherapeutic approaches.

**Table 2:** c-FLIP-mediated immunoregulatory functions.

Cellular FLICE-inhibitory protein (c-FLIP)				
IECs	Biological functions	Isoform	Hypothesized mechanism	Reference
Tregs	↑ Survival	Unknown	CD95-mediated apoptosis-independent mechanisms	(56)
T cells	↑ Proliferation ↑ Proliferation ↑ IL-2 production ↑ Survival and growth ↑ Proliferation ↑ Survival ↑ Survival	c-FLIP_L_ c-FLIP_L_ c-FLIP_L_ p43-FLIP p43-FLIP p22-FLIP c-FLIP_L_ c-FLIPs p22-FLIP	Activation of NFkB and ERK-mediated signaling pathway following the recruitment of TRAF1, TRAF2, RIP, and Rif-1 NFkB and ERK activation due to PI3K signaling pathway Death receptor ligation-independent interaction with Caspase-8, p43FLIP formation and NFkB and ERK activation Interaction with Raf1, TRAF2, and RIPK1 Direct interaction with IKK complex and increase of NFkB activity IKK kinase activation mediated by TAK1 kinase associated with c-FLIP ubiquitin binding function	(57) (58) (59) (59) (60) (61)
Monocytes	↑ MDSCs differentiation	Unknown	Upregulation of immunosuppressive molecules associated to NFkB activation and c-FLIP nuclear translocation	(53)
MDSCs	↑ Survival	Unknown	Unknown	(56)
B cells	↑ Survival	p22-FLIP	Direct interaction with IKK complex and increase of NFkB activity	(60)
DCs	↑ Survival	Unknown	Direct interaction with IKK complex and increase of NFkB activity	(60)

## The Signal Transducer and Activator of Transcription

The signal transducer and activator of transcription (STAT) protein family consists of seven members, which are encoded by distinct genes: STAT1, STAT2, STAT3, STAT4, STAT6, and the closely related STAT5A and STAT5B. The STAT proteins stand out for dual roles: they both transduce signals through the cytoplasm and operate as transcription factors in the nucleus (65, 66). The activation of STAT proteins often involves a ligand-receptor interaction. The STAT activators, which are specific cytokines and growth factors, bind to their cognate receptors leading to the recruitment and phosphorylation of Janus Kinase (JAK) family kinases (JAK-1, JAK-2, JAK-3, and TYK2) (65, 67, 68). Following phosphorylation of specific tyrosine residues in STAT proteins by activated JAKs, STATs form stable homodimers or heterodimers with other STAT proteins through reciprocal phosphotyrosine-SRC homology 2 (SH2) domain interactions. After this post-translational modification, STAT dimers translocate to the nucleus where they bind to consensus DNA sequences and transactivate specific target genes involved in the control of proliferation, differentiation, and apoptosis processes (66, 69).

STAT3 responds to a wide variety of extracellular polypeptide ligands such as IL-6, IL-10, IL-23, IL-21, and IL-11, as well as to leukemia inhibitory factor (LIF) and oncostatin M (OSM) (66, 70, 71). Activated STAT3 protein lead to the transcription of various downstream target genes such as IL-17, IL-23, BCL-X_L_, BCL-2, MCL1, cyclin D1 (CCDN1), c-MYC, and vascular endothelial growth factor (VEGF) that control essential pro-carcinogenic, inflammation-associated networks during cancer progression (66, 67, 71, 72). Indeed, the STAT3 constitutive activation has been reported in nearly 70% of solid and hematological tumors, including multiple myeloma, several lymphomas and leukemias, head and neck cancer, breast and prostate cancer, ovarian carcinoma, melanoma, renal carcinoma, colorectal carcinoma, and thymic epithelial tumors (73). Interestingly, in the cancer microenvironment a STAT3 activation loop between tumor cells and non-transformed cells including immune cells can be established (70) (**Table 3**). In fact, cancer-derived soluble factors can regulate the development, accumulation, and functions of several tumor-infiltrating leukocytes such as tumor-associated macrophage (TAM), M2 macrophage, T-helper 1 (Th1), follicular helper T (Tfh), Tregs, and DCs by switching on a STAT3-dependent signaling (70).

**Table 3:** STAT3-mediated immunoregulatory functions.

Signal transducer and activator of transcription 3 (STAT3)				
IECs	Biological functions	Hypothesized mechanism	Reference
DCs	↓ Maturation ↓ IL-12 release	STAT3-mediated upregulation of S100A9 Signaling mediated by IL-6	(74) (75)
Macrophages	↓ M1 polarization ↑ M2 polarization	Persistent and dominant STAT3 activity	(76)
T cells	↑ Th17 development ↑ Treg induction	Transcriptional regulation of RORγt and RORα Direct activation of FoxP3 transcription	(77, 78) (79, 80)
MDSCs	↑ Immunosuppression ↑ Accumulation	Induction of *Arg1* transcription and Arg1-mediated immunosuppression STAT3-mediated upregulation of S100A9	(81) (74)

For instance, STAT3 orchestrates the fine-tuning activity of macrophages in cancer. In the tumor microenvironment, a dominant and persistent STAT3 activity efficiently suppresses M1 macrophage polarization, dampening cytotoxic and proinflammatory functions (82) and favoring the accumulation of M2-polarized TAMs (83), that contributes to tumor immunoescape releasing pro-angiogenic molecules (e.g. VEGF, C-C motif chemokine ligand 2, CCL2), essential tumor-growing factors (e.g. epidermal growth factor, EGF), and immunosuppressive mediators (e.g. IL-10, transforming growth factor, TGF-β) (70, 76). Notably, STAT3 activity in TAMs seems to promote tumorigenesis and therapeutic resistance by supporting the persistence of a population of cancer cells with increased tumorigenic potential, known as cancer stem/initiating cells (84). Not surprisingly, the abrogation of STAT3-associated signaling pathways inhibits M2 polarization and restrains tumor growth (85).

Moreover, STAT3 signaling plays a pivotal role in the conversion of monocytes into functional MDSCs in cancer setting (86, 87). MDSCs represent a heterogeneous population of not fully differentiated myeloid cells that exhibits potent immunosuppressive functions in the tumor microenvironment (88–90). MDSCs inhibit the anti-tumor immunity through both direct and indirect mechanisms. Indeed, MDSCs act either directly by consuming and/or converting essential metabolites to active cytotoxic products (e.g. reactive-oxygen species, ROS) and by expressing inhibitory receptors that inhibit T cell fitness, or indirectly by switching off the anti-tumor immune response through the release of several soluble mediators able to recruit and activate other immunoregulatory cell populations such as Tregs (89, 91). For instance, Vasquez-Duddel and co-authors reported that p-STAT3 is able to bind different sites on the *arginase-1* (*ARG1)* promoter to favor its transcription (81). ARG1 is a hallmark of MDSCs. Indeed, MDSCs consume environmental arginine through ARG1 activity, producing ornithine and urea. Low levels of arginine together with high concentration of catabolites trigger T cell proliferation arrest and functional impairment by downregulation of the CD3 ζ chain in the T cell receptor (TCR) complex (92–94). In the light of these premises, a unique STAT3-dependent expression of ARG1 in a subset of pancreatic cancer patient-derived monocytes, which exhibit immunosuppressive properties, was recently identified (95). Finally, the STAT3-dependent overexpression of S100A8 and S100A9 proteins, which are important mediators in cancer-induced inflammation, prevents also the normal differentiation of myeloid progenitor cells, promoting their conversion in functional MDSCs (74, 96).

Cancer-induced factors manipulate also DCs differentiation by a STAT3-dependent signaling pathway. Notably, IL-6/STAT3 conditioned monocyte-derived DC (MoDC) showed an incomplete ability to induce cancer-related cytokines production by antigen-specific Th cells. IL-6/STAT3 signaling attenuated IL-12 production by MoDC impairing IFN-γ secretion from CD4^+^ T lymphocytes (75). DC differentiation can be completely restored using repressing approaches based on STAT3 inhibition, which abrogated the negative effects of tumor-derived factors on myeloid-cell differentiation (97, 98). All these data confirm the crucial role of STAT3-dependent signaling on the biology of cancer-reprogrammed myeloid cells.

Similarly, STAT3 drives the acquisition of pro-tumor functions of adaptive immunity. STAT3 triggers Th17 development through the regulation of retinoic acid receptor-related orphan receptor-γt (RORγt) and RORα, which are the master transcription factors driving the lineage commitment to Th17 cells (77, 78). Indeed, *in vivo* experiments showed that STAT3 overexpression supports Th17 cell maturation and differentiation *via* IL-17 production (99, 100). In particular, the Fam64a protein, which promotes STAT3 activity through modulating its DNA-binding ability, plays an essential role in the differentiation and development of Th17 cells in cancer-associated colitis (101). STAT3 is also a crucial tenet of tumor-induced Tregs biology. The frequency of tumor-infiltrating Tregs is now a remarkable predictor of attenuated survival in cancer patients, confirming the impact of this lymphocyte subset on sustaining tumor progression (102, 103). Interestingly, STAT3 controls the expression of Forkhead box P3 (FOXP3) protein in Tregs, binding directly the promoter of the gene coding for this lineage-specification transcription factor. This mechanism is critical in maintaining an immune suppressive state of tumor microenvironment from functionally Tregs activated by a FOXP3-dependent manner (79, 80, 104).

For many years, STAT3 impact on cancer immunity was exclusively focused on its activity as a nuclear localized transcription factor. However, recent data suggest that STAT3, as a moonlighting protein, can regulate different molecular pathways based on its cellular localization (8). Indeed, STAT3 has also nuclear-independent functions in the mitochondrial compartment where it controls different biological processes. Mitochondrial STAT3 (mitoSTAT3) interacts with different proteins, including complex I of the electron transport chain (ETC), mediator of mitochondrial permeability transition pore (mPTP), and cyclophilin D. Such interactions regulate various processes such as the production of ROS and mitochondrial transcription and oxidative phosphorylation (105, 106). MitoSTAT3 not only influences complex II and complex V (ATP synthase) activity (107, 108) but it also leads to Ras-induced transformation (6). These mitoSTAT3-dependent functions are mediated by the phosphorylation state of serine 727 (Ser^727^) (105, 109), located in the carboxyl-terminal transcriptional-activating domain (65). Interestingly, STAT3 phosphorylation on Ser^727^ induces the protein translocation into mitochondria without any structure modification (107, 108). In fact, STAT3 mitochondrial translocation is mediated by interactions with different partners such as heat shock protein 22 (HSP22), gene associated with retinoic and interferon-induced mortality 19 (GRIM-19) or translocase of outer mitochondrial membrane 20 (TOM20) (110–112). In breast cancer settings the substitution of Ser^727^ for alanine or aspartate in mitoSTAT3 affects tumor development in an independent manner of STAT3 nuclear activity. Indeed, cells expressing mutated mitoSTAT3 (S727A) display a slower tumor growth, a decreased complex I activity of the ETC, and an increased ROS accumulation under hypoxia; on the contrary, cancer cells expressing mutated mitoSTAT3 (S727D) show an enhanced tumor progress as well as an increased activity of complex I (6).

Therefore, the subcellular localization of STAT3 directly guides canonical and non-canonical pathways that are involved in tumorigenesis and tumor-induced reprogramming of immune system, highlighting STAT3 as a primary target to build up effective immunotherapeutic strategies.

## Heat Shock Proteins

Heat shock proteins (Hsps) are conserved mediators, which drive essential pathways in promoting protein folding, protein trafficking, and protein complex assembly/disassembly (113, 114). Moreover, Hsps are involved in determining the fate of misfolded proteins by either refolding them or delivering them to the ubiquitin proteolysis pathway for their degradation (115). Currently, Hsps are classified according to their molecular size as Hsp27, Hsp40, Hsp60, Hsp70, and Hsp90. Initially, Hsps were exclusively identified as intracellular factors. However, several reports demonstrated Hsps to be expressed on cell surface as well as secreted in the extracellular space in response to stress stimuli (116). Therefore, Hsps can be considered as moonlighting molecules due to their secondary roles beyond their primary function as chaperones. The surface expression and the release of Hsps can strongly modulate several immune responses. Therefore, it is unsurprising that Hsps are implicated in various pathologies, including cancer. Several Hsps are highly expressed in different types of carcinomas and they are actively involved in promoting the proliferation and differentiation of tumor cells, as well as in the induction of metastatic processes (117, 118). Moreover, the level of circulating Hsps has been already validated as reliable biomarkers of stage and aggressiveness of specific types of cancer. For instance, increased expression of Hsp27 or Hsp70 has been associated with poor outcome in osteosarcoma, gastric carcinoma, breast cancer, and leukemia patients (119). Furthermore, the expression levels and the cellular localization of Hsp60, which is mainly found at mitochondrial level, are altered in different tumor settings (120, 121). Similarly, the high-molecular weight Hsp90 chaperone is also an important regulator of tumor progression and several inhibitors have shown to exert very promising effects against carcinogenesis (122, 123). More importantly, Hsps are strictly involved in influencing the cross-talk between cancer and immunity (**Table 4**) (117). For instance, extracellular Hsps are involved in the activation of DCs promoting the cross-presentation of cancer peptides (117, 124). Tumor antigens can be recognized and uptaken by the antigen-presenting cell (APC) as a complex with Hsps, identified as “Hsp-peptide complex,” which could be either internalized by CD91-mediated endocytosis or bound by Hsps receptors, such as SREC-I (Scavenger receptor expressed by endothelial cells) and LOX-1 (lectin-like oxidized low-density lipoprotein receptor-1) expressed on cell surface, resulting in APC activation (130). This APC activation leads to innate and adaptive immunological responses against cancer cells (117). LOX-1 mainly binds to Hsp60 and Hsp70 and SRECI binds to a wide range of Hsps, including Hsp60, Hsp70, Hsp90, Hsp110, gp96, and Glucose-regulated protein 170 (GRP170) (130, 131). Moreover, the incorporation of tumor-derived peptides in the Hsp-peptide complex prevents its degradation. On the other hand, in the context of inflammatory diseases, microbial Hsp70 has been shown not only to induce tolerogenic DCs but also to promote a suppressive phenotype and activity in MDSCs and monocytes (125, 132). In addition, such DCs could induce Tregs, contributing to reduce inflammatory responses. Furthermore, extracellular interaction with Hsp70 downregulates CD86 and MHC class II expression in DCs and inhibits TNF-α production (126, 132). Hsp70 mediates, for example, the MDSC-associated suppressive activity through the activation of STAT3/ERK pathway, leading to the release of IL-10 (128, 132). In addition, Hsp70_450-463_ peptide (TKDNNLLGRFELSG, defined as TKD) can directly interact with NK cells through the C-type lectin receptor CD94, which is involved in NK cell-mediated recognition of HLA-B, HLA-A, and HLA-C alleles (129). Tumor cells expressing TKD on their cell surface are preferentially recognized and lysed by NK cells. Moreover, Hsp70 and Hsp70-peptide TKD induce both proliferative and cytolytic activity in NK cells (133). Gross et al. demonstrated that TDK stimulation induced the upregulation of CD94 and CD56 in primary NK cells, which was associated with an increased cytolytic response against Hsp70 membrane-positive tumor target cells (129). Chalmin et al. reported also that membrane-associated Hsp70 expressed in tumor-derived exosomes (TDEs) is partially responsible for inhibiting tumor immune surveillance by promoting MDSCs suppressive functions (128). It was demonstrated that TDEs released by different tumor cell lines could mediate T cell-dependent immunosuppressive functions of MDSCs, suggesting that the immunomodulatory effects of tumor cells include their ability of inducing functional MDSCs by releasing exosomes expressing Hsp70 (128).

**Table 4:** Hsp-mediated immunoregulatory functions.

Heat Shock Proteins (Hsps)				
IECs	Biological functions	Protein	Hypothesized mechanism	Reference
DCs	↑ Activation and cross-presentation of cancer peptides ↓ Activation ↓ TNF-α production	Extracellular Hsps Hsp70 Extracellular Hsp70	Tumor antigen incorporation in “Hsp-peptide complex” and consequent CD91- or Hsp receptors–mediated internalization Unknown ERK activation and IL-10 production	(124) (125) (126)
Macrophages	↑ IL-6, TNF-α, IL-12, IL-15 release	Hsp60	CD14-mediated signaling and involving p38 mitogen-activated protein kinase	(127)
MDSCs	↑ Suppressive activity ↑ IL-10 release	TED-associated Hsp70	ERK and STAT3 activation	(128)
NK cells	↑ Cytolytic activity against tumor cells ↑ Proliferation	Hsp70 TDK	Interaction with C-type lectin receptor CD94 Upregulation of CD94 and CD56	(129)
T cells	↓ Proliferation ↓ IFN-γ release	Hsp70	Unknown	(125)

Similarly, Hsp60 can be recognized by receptors of both the innate and adaptive immune system triggering either pro-inflammatory or anti-inflammatory responses by promoting DCs survival, the macrophages maturation, and a reduced migration of effector T cells (127).

The immunomodulatory functions of Hsps have led to their classification as “Chaperokines,” thus suggesting a great potential of Hsp-based immunotherapeutic approach for the treatment of cancer. Notably, Mbofung et al. have recently showed that Hsp90 inhibition enhanced cancer immunotherapy by upregulating interferon response genes (134). In detail, the inhibition of Hsp90 with Ganetespib promotes the *in vitro* T-cell-mediated killing of patient-derived human melanoma cells as well as an enhanced *in vivo* anti-tumor response evocated by anti-CTLA4 and anti-PD1 therapy. The therapeutic effect induced by Hsp-targeting has been also validated by Hsp70- and Hsp90-based anti-cancer vaccines, which are able to contrast tumor progression by an antigen specific cell-mediated response (117). Collectively, these findings highlight the relevance of Hsps as potent immunomodulatory molecules involved in several pathways determining the type and intensity of immune response.

## Histone Deacetylases

Histone deacetylases (HDACs) represent a superfamily of enzymes that remove an acetyl group from ϵ-N-acetyl lysine amino acids. The name HDAC implies a specificity for histone proteins. Nevertheless, HDACs are able to deacetylate a wide range of non-histone proteins in different cell compartments (135, 136). Indeed, the acetylation status is a common post-translational modification of more than 1,000 histone and non-histone proteins, including DNA-binding and DNA-repair proteins, transcription factors and chaperones (137–140). Considering their activity, HDACs are important epigenetic regulators of gene expression and are involved in controlling several cellular processes. Thus, the complex activity of HDACs enzymes has been widely studied in the last years, leading to an increasing interest in understanding the complicated biological role of acetylation and post-transcriptional modification. HDAC enzymes have been shown to interact with each other’s and also with a multi-subunit protein complex, which have many non-histone targets, resulting in the modulation of signaling at cellular and systemic level, often with cell-type specific effects (135, 141–143). Thus, the inhibition of a specific HDAC may have context-dependent consequences on cellular functions. Moreover, it has been widely demonstrated that aberrant gene transcription resulting in HDAC overexpression is associated to increased tumor cell proliferation as well as regulation of inflammatory processes (144). Considering different biological processes mediated by HDAC, its potential ability to modulate the immune system offers intriguing therapeutic possibilities (**Table 5**). Both class I (HDAC1, 2, 3, 8) and class II (HDAC4, 5, 6, 7, 9, 10) enzymes are involved in regulating pro-inflammatory responses (152). Indeed, these enzymes act directly on the modulation of gene transcription or indirectly by specific protein interactions, including transcription factors such as NF-κB. In fact, HDAC1 and HDAC2 can interact with NF-κB family proteins, p65 and p50, resulting in a wide-ranging effect on the immune system. These protein interactions downregulate NF-κB-mediated gene transcription (141, 153). Moreover, HDACs influence the balance of inflammatory responses mediated by IL-2, IL-6, IL-8, IL-1β, and GM-CSF by regulating the histone acetylation status of NF-κB and AP-1 (154). HDACs have also been reported to inhibit DCs functions by repressing the acetylation of histone and non-histone proteins, such as STAT-3, with important implications on the effector mechanisms of immunity (145). Furthermore, T cell responses are strongly influenced by HDACs functions that regulate the maintenance of FOXP3 expression in Tregs (146) and control Th1 and Th2 differentiation of naïve CD4^+^ T cells by reducing the hyperacetylation of histones 3 and 4 at the IFN-γ promoter (147, 155). Moreover, Gialitakis et al. reported that HDACs interact with regulators of MHC class II gene activation, acting as molecular switchers that turn off this process (156). Considering the involvement of HDACs in modulating immune responses, the identification of molecules able to inhibit specific HDAC enzymes offers a novel approach to the treatment of immune mediated diseases and tumor. The clinical use of HDAC inhibitors was mainly tested on the treatment of cancer due to their documented antiproliferative activities involving the regulation of gene expression, cell cycle arrest, apoptosis, and anti-angiogenetic effects (157, 158). Nevertheless, several studies have focused on the therapeutic efficacy of these inhibitors in modulating anti-tumor immunity since their ability to enhance tumor antigenicity and, thereby, prevent tumor escape. Indeed, HDAC pharmacological inhibitors upregulate both MHC class I and II, together with CD40, CD80, and CD68 costimulatory molecules, as well as adhesion molecules such as ICAM-1 (Intercellular Adhesion Molecule 1) on tumor cells in the context of acute myeloid leukemia, neuroblastoma, hepatoma, and others, thus promoting natural killer cell-mediated lysis and CD8^+^ T cell response (148, 149, 159, 160). For instance, HDAC inhibition shows a double effect in the context of melanoma. In addition to its capability to reduce tumor cell proliferation, the impairment of HDAC activity results in an increased expression of MHC and costimulatory molecules on tumor cell surface, resulting in promoting T cell activation Abrogation of HDAC6 inhibits IL-10 producing myeloid cells, prompting the expansion of inflammatory APCs (145). However, an opposite effect was reported by using HDAC11 inhibitors, improving IL-10 production by immunosuppressive myeloid cells (161). Furthermore, Wood et al. showed that class I HDAC inhibitors support the overexpression of PD-L1 in melanoma cells enhancing the efficacy of anti-PD-1-mediated anti-cancer immune response (162). All these recent findings highlight the crucial role of HDAC enzymes in modulating the activity of the immune system and, consequently, pave the way for the development of novel anti-cancer therapeutic strategies.

**Table 5:** HDAC-mediated immunoregulatory functions.

Histone deacetylase (HDAC)				
IECs	Biological functions	Hypothesized mechanism	Reference	
DCs	↓ Activation	Repressing acetylation of histone and non-histone proteins, including STAT-3	(145)	
Tregs	↑ Maintenance	Epigenetic-mediated mechanisms	(146)	
T cells	Controlling Th1 and Th2 differentiation ↑ CD8^+^-mediated anti-tumor response	Deacetylation of histones 3 and 4 at the IFN-γ- promoter Upregulation of MHC class I and II, CD40, CD80, CD68, ICAM-1 on tumor cells	(147) (148–151)	

## Stimulator of Interferon Genes

The detection of damage-associated molecular patterns (DAMPs), such as aberrant nucleic acids released from ultraviolet exposition or materials derived from the extracellular matrix following tissue injury, is an essential process for triggering immunity against tumor cells. In 2008, STING (Stimulator of Interferon Genes), has been identified as an essential protein sensor for the recognition of cytosolic DNA (163). The absence of STING leads to a reduced spontaneous anti-cancer immunity, thus suggesting the central role of such protein in controlling anti-cancer immune responses and the consequent targeting for cancer immunotherapy. Among the immune cells specialized to detect danger signals, DCs assume a central role due to their ability to internalize, process, and present antigens to T cells. DCs can detect danger signals through their expression of pattern recognition receptors, allowing these cells to recognize pathogens as well as endogenous signals released by dying cell, including DNA, which is a strong immune stimulatory molecule widely used as vaccine adjuvant to drive immune responses (164). STING is a transmembrane component of the endoplasmic reticulum (ER) and it is a crucial factor for the production of type I IFN in fibroblasts, macrophages, and DCs in response to cytoplasmic double-stranded DNA (165). Considering that STING does not share any homology with the well-known immune receptors, it has been classified as a novel family of proteins involved in immune signaling triggered by cytosolic DNA (166), including also the cyclic GMP-AMP synthase (cGAS) (167). In detail, following the binding to cytosolic DNA species from viruses, bacteria, or self-DNA, cGAS catalyzes the production of a cyclic dinucleotide called cGAMP (cyclic GMP–AMP), which in turn is recognized by STING (168, 169). STING activation leads to the phosphorylation of interferon regulatory factor 3 (IRF3) and NFκB followed by the subsequent induction of cytokines and proteins, such as the type I IFNs (170). Notably, Woo et al. demonstrated that the spontaneous CD8^+^ T cell priming against tumors was defective in mice lacking STING in the context of melanoma (171). Accordingly, recent findings reported that the activation of STING and the subsequent expression of type I IFN is associated with a CD8^+^ T cell-mediated anti-tumor response (172). Considering that the IFN-β is mainly produced by DCs in the tumor microenvironment, different authors have hypothesized a model in which DCs can uptake and sense DNA-associated entities released by dying cells to induce type I IFNs secretion. These cytokines act in a paracrine or autocrine manner to enhance DCs cross-presentation activity and T cell activation. Furthermore, loss of STING activity in DCs impairs the generation of follicular Th cells and plasma cells, as well as anti-nuclear Abs in a model of systemic lupus erythematosus (173, 174). In a mouse model of glioma, CD11b-expressing, brain-infiltrating leukocytes are the main source of type I IFNs and tumor-bearing mice knockout for STING showed lower expression of IFNs, an increased amount of immature myeloid suppressor and regulatory T cells, and a decrease of IFNγ-producing CD8^+^ T cells, associated to a poorer survival rate, compared to wild-type tumor-bearing mice (175). Accordingly, CD4^+^ and CD8^+^ T cells that received direct type I IFN signals showed a lower degree of regulatory activity and increased levels of anti-tumor activity, respectively (176). These findings suggest that the STING pathway should be re-programmed for improving anti-cancer immunotherapy.

Besides its role in the physiological host defense, several gain-of-function mutations in the STING-encoding *TMEM173* gene have been reported as causative mutations in inflammatory diseases, including STING-associated vasculopathy with onset in infancy (SAVI), systemic lupus erythematosus–like syndromes or familial chilblain lupus (177–180). These mutations are responsible for the constitutive activation of STING signaling in absence of the ligand biding (179, 181). Recently, Wu et al. reported that some gain-of-function induced a chronic ER stress and unfolded protein response (UPR), leading T cells to become hyperresponsive to T cell receptor signaling–induced ER stress and the UPR, with the consequent cell death by apoptosis (182). The constitutive activation of STING has been shown to disrupt calcium homeostasis in T cells. Accordingly, acute stimulation of mouse cells with STING agonists triggers a rapid increase in intracellular calcium (182, 183). The intrinsic T cell priming effect is mediated through a STING functional region of latest discovery called “the UPR motif,” which is distinct from previously known domains responsible for type I IFN signaling. Therefore, a critical IFN-independent function of STING that regulates calcium homeostasis, ER stress, and T cell survival has been unveiled enlisting STING as a moonlighting protein. The new function of STING signaling in balancing T cell life and death decisions has broad implications on immune and tissue homeostasis and, consequently, in the identification of novel therapeutic targets regulating the immune responses also in the context of tumor diseases. The UPR motif is evolutionarily conserved in most animal phyla and is distinct from the previously characterized constans, co-like, and TOC1 (CTT) domain required for IFN signaling (182). Wu et al. also demonstrated that low-dose of a STING agonist (DMAXX) primes wild-type T cells for cell death after CD3/CD28 treatment, resembling the gain-of- function mutant phenotypes. Interestingly, the disruption of calcium homeostasis seems to be mediated by the translocation of STING from ER to Golgi following its activation (182). Recently, Srikanth et al. demonstrated that the ER calcium sensor STIM1 moonlights as an ER anchor for STING (184). Ectopical expression of the STING-anchoring domain, STIM1, is able to reduce ER stress and cell apoptosis in STING mutant cells. Whether STING directly regulates ER calcium homeostasis through STIM1 or other components of the calcium signaling pathway is still unknown.

The involvement of STING in regulating immune responses represents a strong rationale for its targeting in immunotherapy. Several studies based on STING agonist in combination with immunotherapy show promising results. DMXAA treatment was found to control tumor progression in a rat mammary carcinoma as well in different tumor mouse models (185). Unfortunately, the Phase III trials in non-small-cell lung cancer patients did not confirm the anti-tumor effect of DMXAA in cancer patients (186). More recently, cGAMP, the natural STING ligand, appeared to negatively influence cancer progression in different models including melanoma and colon cancer, by promoting an antigen-specific CD8^+^ T response (176). Importantly, Woo et al. demonstrated that in STING-deficient mice the spontaneous CD8^+^ T cell priming against tumors is defective, and the therapeutic effect of CTLA-4 and anti-PD-L1 treatment is completely abrogated (171). Thus, the manipulation of STING signaling and the possibility to design cGAMP isomers with an enhanced ability to induce type I IFN secretion can be harnessed in the context of combination therapies obtaining more potent anti-cancer immune responses (187, 188).

## Type 2 Transglutaminase

In the recent years, the multiple roles played by type 2 transglutaminase (Tgase2) have been widely described defining it as a multi-faceted protein even with opposite activities. Tgase2 is involved in both cell growth and programmed death playing an important role in physiological and pathological conditions. Tgase2-related research was mainly focused on the enzymatic nature of this protein and the catalyzed reaction, until the publication of crucial discoveries (189). In ‘80s, the pioneering studies of Haddox et al. and Fesus et al. demonstrated the role of Tgase2 in cell growth and programmed cell death (190, 191). Furthermore, Nakaoka et al. described that Tgase2 can bind to membranes participating in receptor signaling like a G-protein and may be a component of a complex regulatory network in which GTP binding switches its canonical function of transamidating enzyme to receptor signaling activity (192). The Tgase2 transamidating and GTP-ase activities have shown to be regulated by the presence of calcium and GTP (189) and the GTP binding makes the enzyme unable to catalyze the reaction of transamidation. Conversely, increased concentration of calcium reduces the Tgase2-binding affinity for GTP inducing the exposure of the active site for transamidation reaction. The different activities mediated by Tgase2 can be correlated to the cellular localization of the enzyme. Tgase2 is mainly expressed in the intracellular compartment but the protein has been detected also in the extracellular space within exosomes or associated with proteins of extracellular matrix, thus influencing several and even opposing processes including tissue stability, angiogenesis, proliferation and differentiation, death and autophagy, cell adhesion and migration (193). Many of these functions seem to be linked to additional scaffolding activities possessed by Tgase2 that are not related to transamidating or GTP-binding and hydrolysis activity. Among these, the ability of Tgase2 to act as protein kinase and protein disulfide isomerase has been proposed (194, 195). The high number of biological processes involving Tgase2 has been ascribed to several events, including different conformations and mutant forms of the protein, or through the modification of intracellular proteins such as NF-κB activity by polymerization of IkB (189, 196, 197). The opposite effect of Tgase on cell functions might be related to the molecular state of cells, which could lead to different cell responses. An emerging hypothesis ascribes the multiple roles of Tgase2, especially the pro-proliferative activity, to its ability to translocate into the nucleus and regulate gene expression (189, 198). Interestingly, Tgase2 has been shown to play a crucial role in promoting apoptotic death in cancer cells induced by photodynamic therapy (PDT) (199). Indeed, PDT led to the release of cytochrome c and apoptosis inducing factor (AIF) by mitochondria, resulting in caspase-dependent and -independent apoptosis, respectively. Released AIF translocated to the nucleus and, together with the caspase-dependent pathway, induced apoptotic cell death. Both the caspase cascade and the activation of AIF following PDT are mediated by Tgase2 activation, triggering the calpain-induced Bax (Bcl-2-like protein 4) translocation. Therefore, Tgase2 can be enlisted as a promising therapeutic target for the treatment of cancer. Moreover, Tgase2 is involved in all stages of tumor biology (200). The molecular mechanisms of action of Tgase2 are strictly affected by its expression, activity, cellular localization, and specific cancer setting. However, Tgase2 has been reported as both a potential tumor suppressor and a tumor-promoting factor and, therefore, its role during cancer evolution is still controversial. Indeed, Tgase2 overexpression and increased activity have been frequently associated with cancer, stem cells’ survival, inflammation, metastatic spread, and drug resistance (201). For instance, Fisher et al. showed that Tgase2 knockdown or inhibition resulted in a reduced EMT, migration and invasion in the context of skin cancer, highlighting that these pro-tumoral mechanisms were mediated by the GTP binding activity of Tgase2 (202). These findings suggest the inhibition of Tgase2 GTP binding/G-protein activity as a potential therapeutic tool to reduce skin tumor survival. On the other hand, Tgase2 expression and activity correlate with cancer cell chemosensitivity and drug-induced apoptosis and the use of inducers of Tgase2 transamidating activity seems to inhibit tumor cell plasticity and invasion. Moreover, all-trans retinoic acid (ATRA), which is a potent inducer of Tgase2, is commonly used for the treatment of promyelocytic leukemia (203). These data clearly indicate that Tgase2 could have several and even opposite effects in tumor biology. The well-known involvement of Tgase2 as inflammation inducer allows to develop innovative strategy to enhance cancer immunotherapy. Several molecules implicated in inflammatory processes, including cytokines and growth factors have shown to induce Tgase2 expression and transamidating activity. As already reviewed by Brown et al., canonical and non-canonical NF-κB signaling assume a key role in regulating Tgase2 function (204). Therefore, the target genes regulated by NF-κB, such as TNF-α, IL-1 and IL-6 are strong inducing-factors of Tgase2 expression (205). Furthermore, TGF-β, generally abundantly secreted in the tumor microenvironment, is able to induce Tgase2 expression and activity through a mechanism mediated by Mothers against decapentaplegic homolog proteins (SMADs) and NF-κB at transcriptional level in the context of ovarian cancer (206). Interestingly, the activation of Tgase2 connected to TGF-β and SMAD signaling is involved in both endothelial tubule formation and tubule loss. Tgase2 inhibitors have shown to prevent tubule formation and such phenotype can be restored by exogenous TGF-β1. Moreover, endothelial cells lacking Tgase2 expression are unable to form tubules and display a strong down-modulation of SMAD signaling compared to the wild-type counterpart (207). Several reports have recently shown the role of Tgase2 in mediating immune responses by DCs and adaptive immunity (**Table 6**). Kim et al. showed that naïve T cells displayed a low expression of Tgase2, which increased following activation (208). The absence of Tgase2 led to a strong inhibition of T cell proliferation by mitigating CD69 and CD25 expression together with IL-2 and IFN-γ secretion, presumably due to a reduction of NF-κB activation. Moreover, splenic T cells isolated from Tgase2-knockout mice immunized with tumor lysate-loaded wild-type DCs and re-exposed *in vitro* to the same antigen, show a strong reduction of memory CD8^+^ T cell generation, indicating an additional role of Tgase2 in memory T cell generation (208). On the other hand, the expression of Tgase2 in cancer cells was recently shown to mediate mechanisms of resistance to immunotherapy (209). In a mouse model of pancreatic ductal adenocarcinoma (PDA), the inhibition of CXCR4 overcomes the resistance to immunotherapy. PDA cancer cells exhibit an extracellular complex that include CXCL12, the ligand for CXCR4, which is expressed by cancer associated fibroblasts (CAF). PDA tumors lacking Tgase2 do not present the CXCL12-based barrier and therefore they are effectively infiltrated by T cells after anti-PD-1 treatment (209). These data suggest a role of Tgase2 in cancer cells in the mechanisms of immune escape by capturing the immunosuppressive chemokine released by CAFs. Many studies have reported an increased expression of Tgase2 in different cancer cell types that are resistant to chemotherapy and/or immunotherapy (200, 201). Furthermore, the down-modulation of Tgase2 enhance the therapeutic efficacy of anti-cancer treatment in distinct mouse tumor models (200, 201), thus supporting the hypothesis of a crucial involvement of such protein in tumor progression and immune escape. Due to its enzymatic and non-enzymatic functions, Tgase2 regulates several physiological processes, such as apoptosis, differentiation, inflammation, fibrogenic reactions as well as it operates modulating cancer to immunity crosstalk; thus, potential Tgase2-targeting approaches should be developed after a careful consideration.

**Table 6:** Tgase2-mediated immunoregulatory functions.

Type 2 Transglutaminase (Tgase2)			
IECs	Biological functions	Hypothesized mechanism	Reference
DCs	Modulation of DC-T cell interaction	Regulation of cell-to-cell contact between DCs and T cells	(208)
T cells	↑ Proliferation ↑ CD69, CD25 Expression ↑ IL-2, IFN-γ release Regulation of memory T cells ↓ T cell tumor infiltration	NFkB-mediated signaling Unknown Induction of CXCX12 expression on tumor cells that inhibits T cell infiltration	(208) (209)

## Glyceraldehyde-3-Phosphate Dehydrogenase

Glyceraldehyde-3-phosphate dehydrogenase (GAPDH) is a well-known enzyme that catalyzes the reversible oxidative phosphorylation of glyceraldehyde-3-phosphate in the presence of inorganic phosphate and nicotinamide adenine dinucleotide (NAD). This reaction is a key process of the glycolytic pathway for the generation of ATP. Recent studies have clearly revealed multiple unexpected functions mediated by GAPDH as moonlighting protein. Cytosolic GAPDH can regulate gene expression through mechanisms involving mRNA stability (210) and microtubules organization (211). As already reviewed by Sirover, several studies clearly demonstrate that GAPDH is not only a cytosolic protein, but it can be also localized in the cell membrane, nucleus, polysomes, ER, and Golgi (5). The subcellular localization is strictly related to its multiple functions as regulator of iron metabolism, membrane trafficking, histone biosynthesis, the maintenance of DNA integrity and receptor-mediated cell signaling (5). Furthermore, GAPDH deregulation has been detected in several cancer types such as lung, renal, breast, gastric, and pancreatic cancer, thus suggesting a potential role in tumor progression (212–214). Despite the fact that different studies investigated the multiple functions of intracellular GAPDH, extracellular GAPDH, that would seem to play an important role in immune responses, is still poorly studied. Takaoka and co-authors demonstrated that exogenous GAPDH strongly suppressed neutrophil lung infiltration in a mouse model of lipopolysaccharide (LPS)-induced sepsis-related, severe acute lung injury (215). Similarly, Raje et al. reported that GAPDH is expressed on the macrophage surface, supporting the function of transferrin receptor (216). In addition, GAPDH is involved in the anti-infective peptide (LL-37)-induced innate immune response in monocytes (217). These observations strongly highlight that GAPDH might act on macrophage fitness and activity and therefore, GAPDH plays a key role on controlling macrophage biology. Likewise, Nakano et al. showed that GAPDH strongly suppressed cell adhesion, spreading, and phagocytic function of LPS-stimulated macrophages, while it did not affect their viability (218). The treatment with exogenous GAPDH significantly reduced the release of TNF-α, whereas in the same experimental setting it induced IL-10 production in a dose-dependent manner (218). Furthermore, GAPDH induced the upregulation of both nitric oxide synthase (NOS2) and Arg1 in macrophages, which are crucial and well-known immunosuppressive enzymes overexpressed in myeloid regulatory cell subsets such as M2-polarized macrophages and MDSCs (218). According to these findings, a novel moonlight function of GAPDH during inflammatory processes was proposed. Indeed, extracellular ATP may induce activated macrophages to release GAPDH, which promotes in turn the immunosuppressive M2-like phenotype to inhibit inflammatory responses by modulating intracellular NAD/NADH balance (219).

## Calreticulin

Calreticulin (CRT) is a well-known moonlighting protein, which is generally localized in the lumen of ER. In this cell compartment, CRT is involved in several biological functions, including the regulation of calcium homeostasis, protein folding promotion, and the regulation of antigenic peptides loading of class I MHC molecules (220). Recently, the relevance of CRT in mediating immunomodulatory processes was uncovered (**Table 7**). Surprisingly, these unexpected functions are carried out in the extracellular space. Soluble CRT has shown to accumulate in the serum of patients with rheumatoid arthritis or systemic lupus erythematosus, which could lead to macrophage activation and antibody secretion (223, 227). The treatment of mouse macrophages with recombinant CRT induces IκBα degradation followed by NF-κB and MAPK activation, inducing the release of pro-inflammatory cytokines such as TNFα and IL-6 (224). Furthermore, the expression of CRT on extracellular cell surface of DCs acts as DAMPs, which in turn allows the effective DC maturation (221). Moreover, the induction of apoptosis in cancer cells by several anti-cancer agents induces an ER-stress response, resulting in the translocation of CRT on cell surface. This phenomenon, together with the release of HMGB1, promotes the phagocytic uptake of apoptotic cells by DCs and the consequent Toll-Like Receptor 4 (TLR4)-dependent antigen capture and processing (222). Interestingly, the treatment of cancer cells with TRAIL, that induce the assembly of DISC (death-inducing signaling complex) and the proteolytical processing of Caspase 8, have reported to induce the exposure of calreticulin and immunogenic cell death (222). Since numerous types of immune cells such as NKs, monocytes, and T cells express TRAIL, the role of CRT in tumor immunity could be relevant for developing novel approaches of immunotherapy. Bruyn et al. demonstrated a strong co-localization of CRT with TRAILR2 in melanoma cells (228). Moreover, Gardai et al. discovered that the surface of pre-apoptotic cells is enriched of CRT-based complex characterized by high levels of phosphatidylserine, a well-known apoptotic marker (229). On the other hand, surface CRT on viable cells is associated with CD47, an inhibitor of phagocytosis (230). Thus, the induction of apoptosis leads to the dissociation between CRT and CD47 and CRT associates to phosphatidylserine lipid rafts. The balance between pro-apoptotic and anti-apoptotic signals mediated by CRT and CD47 was detected in several cancer cells (228, 230, 231). Interestingly, Wang et al. demonstrated the ability of CRT to act as tumor-associated antigens, triggering a specific anti-tumor immune response mediated by DCs and T cells (223). Therefore, genetically engineered DCs to express CRT constitutively, increased T cell activation. Accordingly, in a previous report, CRT has shown to promote tumor lymphocyte infiltration and enhance the efficacy of immunotherapy, ascribing such effect to the upregulation of adhesion molecules such as ICAM-1 and VCAM1 in tumor endothelium (225). In a recent retrospective study, Kasikova et al. reported the potential impact of CRT on immune responses and the possibility to use CRT as prognostic factor in the context of primary and metastatic high-grade serous carcinomas (HGSCs) (226). The authors demonstrated that the exposure of CRT on the surface of HGSC cells triggers a local immune response mediated by Th1-polarized lymphocytes. On the other hand, the release of mutant forms of CRT, which are normally depicted in patients with myeloproliferative neoplasms (MPNs), is able to prevent the anti-tumor immunity (232). These mutations give rise to a CRT variant lacking the ER retention signal and able to bind the thrombopoietin receptor, leading to the activation of JAK2. Consistently, MPN patients characterized by aberrant CRT not only display higher plasma levels of soluble CRT than healthy donors but, more interestingly, the mutated protein significantly reduces the phagocytosis of CRT-exposing cells. Indeed, the excess of soluble CRT might saturate CRT receptors on phagocytic cells. Unlike wild-type cells, clones expressing CRT mutants induced elevated levels of soluble CRT, leading to an expansion of MDSCs in the spleen and in peripheral blood in different tumor mouse models, with the consequent failure of anti-tumor treatment (232). Therefore, the presence of mutant CRT could be identified as responsible for some mechanisms of immune escape in several aggressive tumors.

**Table 7:** Calreticulin-mediated immunoregulatory functions.

Calreticulin (CRT)				
IECs	Biological functions	Hypothesized mechanism	Reference	
DCs	↑ Maturation ↑ Phagocytic uptake of apoptotic cells and TLR4-dependent antigen processing ↑ Anti-tumor response	Extracellular cell surface expression acting as a danger associated molecular pattern molecule CRT translocation on the cell surface of cancer cells CRT as tumor-associated antigen promoting DCs activation	(221) (222) (223)	
Macrophages	↑ IL-6 and TNF-α release	IκBα degradation and consequent NFκB and MAPK activation	(224)	
T cells	↑ Anti-tumor response ↑ tumor infiltration ↑ Th1 polarization ↑ Cytotoxic activity	CRT as tumor-associated antigen promoting T cell activation Upregulation of adhesion molecules in tumor endothelium mediated by CRT expression in cancer cells Effect mediated by CRT expression in cancer cells	(223) (225) (226)	

## Cytochrome C

Cytochrome c (Cytc) displays multifunctional properties, not only in mitochondrial metabolism but also as apoptosis regulator, thus it is a full-flagged moonlighting protein (233, 234). These unconventional functions of Cytc drive important steps of tumor progression (235).

Cytc is a small, globular, highly conserved protein with a covalently attached heme group that performs multiple functions and catalytic activities (236). Cytc is localized in the mitochondrial intermembrane space since it is part of the electron transport chain (ETC) (8). In the mitochondrial ETC, Cytc acts as an electron carrier from complex III (cytochrome bc1) to complex IV (Cytc oxidase COX). Cytc-knockout mice die at mid-gestation, when fetal metabolism switches from glycolysis to oxidative phosphorylation, demonstrating that ETC function fully relies on the presence of Cytc (236–238).

In addition, Cytc plays a central role in apoptosis. Indeed, Cytc is released from the mitochondria into the cytosol during cellular stress, because of mitochondrial outer membrane permeabilization (MOMP) (239). In the cytosol, Cytc acts as a signaling molecule and promotes the formation of the heptameric apoptosome by interacting with apoptotic protease-activating factor 1 (Apaf-1). Apoptosome finally activates a caspases cascade signaling that results in apoptotic cell death (240). Cytc is tightly regulated by allosteric mechanisms ad post-translational modifications (PTMs). Under normal conditions, distinct residues of Cytc are modified by PTMs leading to downregulation of mitochondrial ETC flux and adjustment of mitochondrial membrane potential, to minimize ROS production. By contrast, pathologic and acute stress conditions lead to maximum ETC flux, hyperpolarization and excessive ROS generation, and the release of Cytc. In this case, dephosphorylated Cytc in cytosol leads to maximum caspase activation (236). Indeed, Cytc deficiency in cancer cells abrogates apoptosome-mediated caspase activation and contributes to mitochondrial dysfunction, thereby promoting therapeutic resistance and tumor aggressiveness. In detail, Cytc deficiency correlates to the expression of genes typically involved in the tumorigenesis such as c-Myc, NF-kB and Akt. In prostate cancer cells, the activation of c-Myc and NF-kB or inhibition of Akt prevented nuclear translocation of nuclear respiratory factor 1 (Nrf1), a transcription factor that controls the activation of key genes for regulation of cell growth, respiratory chain, heme biosynthesis and mitochondrial DNA transcription and replication (235, 241–243). Decreased nuclear accumulation of Nrf1 and its subsequent loss of binding to the Cytc promoter mediated Cytc deficiency thus modulating caspase activation. All these data suggest that restoring this moonlighting protein functions may overcome therapeutic resistance and cancer aggressiveness and thus Cytc-targeting approaches may be useful to enhance the effectiveness of conventional anti-cancer therapies.

## Claudins

Claudins (Cldn) are key transmembrane proteins within tight junctions (TJs) that promote cell-cell adhesion by forming intercellular strands comprised of different Cldn combinations (244, 245). Cldn are 20–34 kDa proteins and consist of 27 members characterized by a short cytoplasmic N-terminal region, two extracellular loops formed by four transmembrane domains, and a cytoplasmic C-terminal tail (246). One of the large extracellular loops contains charged amino acids to regulate paracellular ion selectivity of anions and cations (247). Two highly conserved cysteine residues within this loop increase the protein stability by the formation of disulfide bond (248). The shorter second extracellular loop favors dimerization process with Cldns on opposing cell membrane through the hydrophobic interactions between conserved aromatic residues (245). These proteins regulate the permselectivity of tight junctions favoring selectively anion or cation permeability by forming highly regulated pores (249). Given this function, and the fact that cell-cell adhesion complexes are altered during metastatic progression, it is not surprising that Cldn expression plays a pivotal role in regulating metastatic progression (250). Indeed, metastatic tumor cells have to overcome different physical barriers to successfully disseminate in a new tissue and, during this process, the communication between tumor cells and stroma is essential (250). Numerous epithelial-derived cancers display altered Cldn expression patterns and some Cldns are now used as good biomarkers to predict patient prognosis (250). For instance, the decrease of Cldn-1 expression was associated with poorer overall survival in lung adenocarcinomas (250). Similarly, decreased Cldn-3 levels in squamous cell lung carcinomas significantly correlate with tumor stage and disease recurrence (251). On the contrary, the involvement of Cldns in prostate cancer is completely different. Indeed, high tumor grade correlates with both a loss of expression of some Cldns such as Cldn-1 and Cldn-7 and increased levels of other protein forms including Cldn-3 and Cldn-4 (252). Especially, Cldn-4 is highly expressed in both primary and metastatic prostate cancer suggesting a potential role of Cldn-4 as a prognostic factor for advanced and metastatic prostate cancer (253, 254). Finally, the expression of Cldn-18 is markedly decreased in gastric cancer and in lung adenocarcinoma (LuAd), where Cldn-18 expression inversely correlates with LuAd patient mortality (255).

Recently, several efforts have been made to deeply understand the role of Cldns during tumorigenesis and metastatization, in which Cldns could play unconventional functions compared to their prototypical roles within TJ complex. In line with this hypothesis, the nuclear localization of Cldn-1 was depicted in both colon cancer tissues and colon cancer-derived liver metastases, whereas Cldn-1 localization in normal human colonic mucosa is exclusively restricted to the basolateral membrane (256). The presence of Cldns at nuclear level is testified also in other cancer settings (257, 258). For instance, studies carried out in human lung adenocarcinoma demonstrated that forskolin, a lip-soluble compound that stimulates the enzyme adenylate cyclase and cyclic adenosine monophosphatase (cAMP), activates specific protein phosphatase, which in turn dephosphorylates Cldn-2 favoring its nuclear localization (259). Once into nucleus Cldn-2 retained the transcription factor ZONAB [zonula occludens 1 (ZO-1)-associated nucleic acid binding protein] and Cyclin D1 to enhance cancer cell proliferation (259). Therefore, it was suggested that Cldns utilize their nuclear localization signal (NLS) sequence or PDZ (PSD-95/Dlg/ZO-1) domain or other mechanisms for their transport to the nucleus, where they act directly as transcription factors or conduct still unknown functions (256).

Interestingly, Cldn expression has also an important impact on tumor suppressor activity. Consistent with recent data in Cldn18^−/−^ knockout mice, the abrogation of Cldn-18 in human LuAd cells completely restrains not only the expression of transcriptional co-activator with PDZ-binding motif (TAZ) and Yes-associated protein (YAP), but also YAP nuclear localization and the transcription of YAP/TAZ target genes. The interaction between Cldn-18 and YAP occurs at sites of cell-cell contact, suggesting the sequestration of p-YAP at TJs (260). Moreover, Cldn-18 inhibited insulin-like growth factor-1 receptor (IGF-1R) and AKT phosphorylation. All these data indicate a tumor suppressor role of Cldn-18 in LuAd, mediated by a regulatory network that encompasses YAP/TAZ, IGF-1R, and AKT (255).

Although the existing data seem to support the interaction between nuclear Clnds and transcriptional regulators to impact gene expression and the regulation of cell adhesion, cell proliferation/cell death, more details on Cldn-associated pathways are needed to understand their non-canonical functions during tumor progression. Indeed, Cldns family as moonlighting proteins induce distinct signaling pathways based on their cellular localization and carry out tumor and metastases regulation by mediating different activities. Therefore, given the importance in tumorigenesis, Cldns could be considered as an ideal target for possible strategies aimed at improving the management of metastatic cancer (250).

## High-Mobility Group Box 1

High mobility group box 1 (HMGB1) is a ubiquitous nuclear protein that is present in almost all eukaryotic cells, in which it plays a critical role in maintaining genomic architecture and stability. It is, however, a multifaceted protein that exists in different isoforms and that can play different functions in the nucleus, cytosol or released outside the cell, bound or not to other molecules (**Table 8**). The ability to interact with other molecules and, on this basis, to gain different biological functions, can be considered the main feature of HMGB1 and it is therefore enlisted as an immune moonlighter. The function of HMGB1 as an alarmin takes place both inside and outside the cell (266). HMGB1 is a 215 amino acid protein, member of the HMGB family proteins HMGB1, HMGB2, HMGB3. Only HMGB1 is expressed ubiquitously and abundantly, whereas HMGB2 and HMGB3 after embryogenesis appear localized to lymphoid and testis tissues or hematopoietic stem cells, respectively (267). HMGB proteins share a highly evolutionary conserved primary structure, comprising two tandem DNA-binding domains (A and B box domains) and a negatively charged acidic C-terminal tail as third domain. Proteins of the HMGB family are non-histone proteins binding chromatin according to conformation but independently from sequence. HMGB1 binds the minor groove of DNA and it was initially defined uniquely as a nuclear transcription regulator that, by facilitating the binding of several regulatory protein complexes to DNA, promotes transcriptional activity (261, 268). HMGB proteins were rediscovered also as nucleic-acid-sensing proteins that bind DNA and/or RNA in the cytoplasm. This DNA/RNA/HMGB interaction has been shown fundamental to mediate foreign nucleic acid recognition by TLR3-9 and RIG-I-like receptors and the subsequent inflammasome activation (267, 269). In the presence of cellular stress, ROS are generated, promoting HMGB1 translocation to the cytosol. Cytosol accumulation of HMGB1 promotes autophagy by enhancing ERK signaling and disrupting Beclin1–Bcl2 complex formation (270). In neutrophils HMGB1 is mostly residing in the cytoplasm, ready to be released in the extracellular space in response to inflammatory stimuli (271). Cytoplasmic accumulation of HMGB1 increases innate immune responses to foreign nucleic acids and promotes autophagy, important for the elimination of viruses and intracellular bacteria (269). HMGB1 can be translocated outside the cells by active secretion in intact living cells or by passive release from necrotic cells. Secretion of HMGB1 does not occur through the conventional endoplasmic reticulum/Golgi pathway, because it lacks a leader peptide: during inflammation it is actively released by immune cells through pyroptosis, necroptosis, apoptosis, NETosis, and secretory lysosomes (272, 273). Extracellular release of HMGB1 may be actively induced by cytokines in cells of the innate immune response such as monocytes, macrophages, DCs, NK cells, endothelial cells, and platelets, and in this location HMGB1 behaves as an inflammatory cytokine (274). When released from necrotic cells, HMGB1 acts as a DAMP, binding to cell surface receptors like TLR2, TLR4, and RAGE, alone or associated to nucleosomes, LPS, RNA, and DNA (261). On the other hand, HMGB1 results tolerogenic when released by apoptotic cells, due to a caspase-dependent oxidation (275).

**Table 8:** HMGB1-mediated immunoregulatory functions.

High-mobility group protein 1 (HMGB1)					
IECs	Biological functions	Post-translational modifications	Hypothesized mechanism	Reference	
DCs	↑ Activation and maturation ↑ activation, maturation, and inflammatory cytokines release ↑ Recruitment ↑ Activation and maturation	Disulfide isoform Disulfide isoform Thiol isoform	DAMP by active secretion by immune cells or passive extracellular release by necrotic cells Binding to TLR2, TLR4, and RAGE, associated or not to nucleosomes, LPS, DNA, and RNA and uptake Dimerization with CXCL12 and binding to CXCR4 Activation and maturation by induction of tumor debris uptake and maturation	(261) (262) (263) (264)	
Macrophages	↑ Recruitment	Thiol isoform	Dimerization with CXCL12 and binding to CXCR4	(263)	
T cells	↑ Anti-tumor response by Th1 polarization ↓ Anti-tumor response by Treg activation		DC activation by induction of tumor debris uptake and maturation	(264) (265)	

Several post-translational modifications of HMGB1 have been highlighted and are of utmost importance to dictate its location and function, especially in terms of regulation of the inflammatory response. HMGB1 contains two nuclear localization sequences (NLS) and two non-classical nuclear export sequences (NES), that allow nuclear accumulation as well as nucleus-cytoplasm transfer mediated by the nuclear import or export complexes. At the steady state, the majority of HMGB1 molecules reside within the nucleus, but post-translational modifications of NLS or NES like acetylation, phosphorylation, and methylation may modify HMGB1 localization (273, 276). Hyperacetylation of the lysines contained in the two NLS by cooperation of histone acetylases (HATs) and HDACs results in HMGB1 nuclear-cytoplasm translocation, eventually followed by secretion. Several signals can induce the acetylation of HMGB1, in many cell types: LPS, TNF, IL-1 beta, type 1 and type 2 interferons (IFN), oxidative stress (277, 278). Serine phosphorylation within the NLS sites allows HMGB1 cytoplasm translocation as well, probably by altering the charge of the NLS sites that destroys the interactions with the nuclear importin proteins requiring positive charged residues in the NLS sequences to function (279). The peculiar cytoplasmic localization of HMGB1 in inactive neutrophils appears dependent on a mono-methylation at lysine 42 causing a decrease in DNA-binding activity and leading to a passive diffusion to cytoplasm (271). HMGB1 is subjected to redox modifications at the level of three evolutionary conserved cysteines, Cys23 and Cys45 in the A-box and Cys106 in the B-box. The redox state of these cysteines dictates different extracellular HMGB1 activities (274, 280). The HMGB1 isoform with all three Cysteines reduced (fully reduced or all-thiol-HMGB1) has a potent chemoattractant activity on leukocytes by dimerization with the CXCL12 chemokine and binding to CXCR4 chemokine receptor (263). Formation of a disulfide bond between Cys23 and Cys45 in mild oxidative conditions, which leaves C106 in the reduced state, generates the disulfide-HMGB1, that fail to bind CXCL12, but it is able to promote the release of inflammatory cytokines through TLR4-MD-2 and RAGE receptors interaction (262, 281). ROS produced by leukocytes induce the terminal oxidation of HMGB1 at all three Cysteines, making the molecule immunologically inactive (275). Several studies have shown that HMGB1 can either promote or contrast tumor growth in both tumorigenesis and cancer therapy (261, 276, 282–284). In many clinical settings, high levels of HMGB1 (both intra-tumoral and circulating) have negative prognostic or predictive value; where extracellular HMGB1 secreted by cancer cells can activate proinflammatory signaling pathways promoting tumor growth, metastasis, angiogenesis, and inhibition of CTL-dependent anti-tumor immunity by activation of Tregs and MDSCs. In addition, by upregulating autophagy, HMGB1 can stimulate drug resistance (265, 285, 286). On the other hand, an increase of circulating levels of HMGB1 following anti-cancer treatments like neo-adjuvant chemotherapy or oncolytic virotherapy has been associated with anti-tumor response and partial remission, suggesting that circulating HMGB1 may be indicating the size of immunogenic cancer cell death (264, 287, 288).

## Moonlighting Proteins: A Complication or An Opportunity in Drug Design?

While at present it is difficult to estimate how abundant moonlighting proteins are, it is evident that moonlighting is not a rare phenomenon. Hundreds of moonlighting proteins are currently known: according to the MoonProt Database (289), a searchable and internet-based resource, the number of moonlighting proteins identified so far exceeds 500, and it is likely that more multitasking proteins will come along in the near future.

Due to its recurrence, awareness of moonlighting phenomenon has an impact on many disciplines, including drug discovery. Indeed, it has been estimated that 78% of the human moonlighting proteins are involved in pathological disorders, highlighting the need for a rational design of compounds targeting moonlighting proteins (290).

While in the past the discovery of the secondary function of a protein occurred merely by serendipity, without linking single function to specific domains, nowadays several attempts to bioinformatically predict and identify multitasking proteins have been proposed (291). Moreover, the combination of computational studies, bioinformatics, and proteomics are believed to synergistically operate in order to provide the accurate mapping and localization of the sites associated with canonical and moonlighting functions, paving the way towards the rational design of molecules interfering with one single activity and not the others. Small molecules that interfere with all the activities displayed by multitasking proteins are likely to suffer from side-effects, and from this point of view moonlighting represents a complication in drug discovery. The development of small molecules able to dissect the different biochemical/functional activities is increasingly gaining ground. In this context, the first aim is to access chemical probes as a means to better understand moonlighting proteins and their multiple functions one at a time, but, more importantly, compounds that are able to interfere with one function and not the other might have a therapeutic potential, given the role played by this cluster of proteins in many diseases, as highlighted above.

While many examples of moonlighting proteins where small molecules able to inhibit the canonical and catalytic activity exist, chemical targeting of the moonlighting function is much more challenging and only few examples have been described so far. Among the proteins playing a pivotal role in cancer immunology and discussed in this review, IDO1 and GAPDH are the ones for which efforts in molecular dissection of the protein multiple functions have led to concrete results in terms of drug discovery, despite still at an early stage. A triazine-based molecule (GAPDS) has been reported that, due to its ability to interfere with the secondary function of GAPDH (*i.e.* GAPDH tetramerization) shows greater toxicity and stronger ability to inhibit cell migration and invasion compared to a pure inhibitor of the GAPDH glycolytic activity (292). Interestingly, as a result of the chemical targeting of the moonlighting function, cytoplasmic levels of GAPDH and tubulin expression are reduced.

On the same note, two small molecules that target the moonlighting function of IDO1 have been reported. The first is VIS351 that positively modulates the ITIMs-mediated function of the enzyme, although not showing any catalytic inhibition. It has been speculated that this compound shifts the dynamic conformational balance towards the ITIMs-favoring folding of IDO1, resulting in the activation of the moonlighting function rather than the catalytic activity (293). On the other hand, PCC0208009 is a compound that seems to inhibit IDO1 catalytic activity and participate in the gene regulation of IDO1 expression, even if the molecular basis for this behavior is not clear (294).

## Concluding Remarks

The number of multifunctional proteins and their newly discovered biological activities has been increasing in the recent years. Indeed, almost 500 moonlighting proteins have been identified and most of them conserved in the evolutionary tree (289, 295). Importantly, moonlighting proteins are not the result of gene modifications, splice variants, or promiscuous enzymes. Moonlighting proteins are in fact the result of evolution, during which proteins acquire additional functions in response to environmental pressure, such as temperature changes, alteration of metabolism, hypoxia, and immune attack (296). Therefore, in pathological conditions, such as cancer, some alterations of protein functions cannot be ascribed only to gene mutations, but also to unpredictable protein-to-protein interactions or unexpected cellular localization of proteins. Once a new function has evolved, it might modify not only the biological process of the cell but also cell-to-cell interactions in the microenvironment. This aspect plays a critical role in manipulating the crosstalk between cancer and immune system. A comprehensive assessment and definition of moonlighting protein networks might be crucial to determine not only cancer prognosis, but also the development of more effective cancer therapies. In this light, several research efforts have been directed towards the identification of compounds that are able to interfere selectively either with the canonical or the moonlighting function of a protein. Despite the development of such molecules is still at an early stage, promising results have been reported, especially for some crucial proteins involved in cancer immunology, such as GAPDH and IDO1. The take-home message of this review is that the anti-cancer immune response may be conditioned by unpredictable functions of immune cells as result of the expression of moonlighting proteins able to induce pro-tumor or anti-tumor effects. The advances in our knowledge on the pervasive role of moonlighters on shaping immune cells during cancer evolution, will pave the way for enhancing the efficacy of cancer immunotherapy.

## Author Contributions

All authors listed have made a substantial, direct, and intellectual contribution to the work and approved it for publication. 

## Funding

This work was supported by the PRIN program of Italian Ministry of Education, University and Research (MIUR, CUP: B38D19000140006), and fondazione Associazione Italiana per la Ricerca sul Cancro (AIRC, Project: 21509) to SU.

## Conflict of Interest

The authors declare that the research was conducted in the absence of any commercial or financial relationships that could be construed as a potential conflict of interest.
